# Phthalate release in leachate from municipal landfills of central Poland

**DOI:** 10.1371/journal.pone.0174986

**Published:** 2017-03-30

**Authors:** Paweł Wowkonowicz, Marta Kijeńska

**Affiliations:** Institute of Environmental Protection - National Research Institute, Environmental Chemistry and Risk Assessment Department, Warsaw, Poland; The Education University of Hong Kong, HONG KONG

## Abstract

Phthalate diesters (PAEs) are used as plasticizer additives to polymer chains to make the material more flexible and malleable. PAEs are bonded physically, not chemically, to the polymeric matrix and can migrate to and leach from the product surface, posing a serious danger to the environment and human health. There have been a number of studies on PAE concentrations in landfill leachate conducted in the EU and around the world, though few in Poland. In the present study, the leachate of five municipal landfills was analyzed for the presence of PAEs. Raw leachate was sampled four times over the period of one year in 2015/16. It was the first large study on this subject in Poland. PAEs were detected in the leachate samples on all of the landfills, thereby indicating that PAEs are ubiquitous environmental contaminants. The following PAEs were detected in at least one sample: Di(2-ethylhexyl) phthalate (DEHP), Diethyl phthalate (DEP), Dimethyl phthalate (DMP), Di-n-butyl phthalate (DBP), Di-isobutylphthalate (DIBP). Out of all ten PAEs, DEHP was the most predominant, with concentrations up to 73.9 μg/L. DEHP was present in 65% of analyzed samples (in 100% of samples in spring, 80% in winter, and 40% in summer and autumn). In only 25% of all samples DEHP was below the acceptable UE limit for surface water (1.3 μg/L), while 75% was from 1.7 to 56 times higher than that value. On the two largest landfills DEHP concentrations were observed during samples from all four seasons, including on a landfill which has been remediated and closed for the last 5 years.

## Introduction

Phthalic acid diesters (PAEs) are used as plasticizers added to polymer chains to make the material more flexible and malleable. PAE production started in 1920 and intensified after 1950 with the increasing usage of polyvinyl chloride (PCV) [1], which was first synthesized in 1931 [2].

Global usage of PAEs during manufacturing and processing of plastic products is estimated at 5–8 million Mg annually [3–4]. The most popular and frequently used as additive plasticizers during the production of flexible polyvinyl (PVC) are di(2-ethylhexyl) phthalate (DEHP) and di-n-butyl phthalate (DBP) [5]. PAEs are also used in polyvinyl acetates, cellulosics and polyurethane resins [2]. The amount of DEHP and DBP in PCV may reach up to 40–60% [5–6].

PAEs are bonded physically, not chemically, to a polymeric matrix and can migrate to the product surface, potentially leaching into the environment [1–2, 6–7]. Many physico-chemical factors, such as temperature, pressure, pH, and presence of solvents, can influence the rate and speed of phthalate migration [2].

Landfilling is still one of the most common techniques to dispose of waste [8–9]; consequently many plastic products (PVC) will find their way to municipal solid waste (MSW) landfills. If landfills do not have adequate environmental protection systems installed (e.g. leachate collection systems), PAEs can be a serious threat to the environment and to human health [3]. An important fact is that acute toxicity of PAEs is believed to be low, but its breakdown products, such as phthalic acid monoesters (PMEs), may possess toxicity to mammals [10]. Also, while DEHP is not considered acutely toxic, once oxidized it becomes more harmful [11] and more toxic [6] than the original compound. Later on in this paper, whenever environmental issues are mentioned, PAEs should be understood as phthalic acid diesters, and compounds resulting from their degradation (including PMEs).

Leachate is mostly generated through penetration of precipitated water into the landfill. PAEs are easily released from waste and can be found in high concentrations in landfill leachate [4, 7]. PAE presence in MSW leachate has been documented in many studies [4, 7, 12–13]. The rate of PAE release depends on many factors such as type of landfill, and phthalate properties. The influence of various factors such as seasonal variations, and landfill size and age, are still poorly recognized [13]. Possible penetration of leachate to ground and surface water can cause water contamination, rendering it unpotable [14].

Phthalates, or esters of phthalic acid, are toxic [15] and ubiquitous environmental pollutants [3, 16], and some of them (e.g. butyl benzyl phthalate (BBP), DBP, DEHP,) are identified as hazardous substances [17]. According to European regulation, DEHP is a priority substance in Europe and its level in surface water should not exceed 1.3 [μg/L] [18]. The EPA (U.S.A.) included PAEs (e.g. dimethyl phthalate (DMP), DEHP, and DBP) on its Toxics Release Inventory (TRI), which consists of chemicals that cause adverse environmental or human health effects. PAEs are on the list “because of their toxicity and the evidence of pervasive human and environmental exposure to them” [19].

PAE usage is regulated in various EU legislative acts. The use of DEHP, DBP and BBP in children’s toys and childcare products is limited to 0.1% of weight [20]. Also, WHO and EU guidance set the maximum safe concentration of DEHP in drinking water as 8 μg/L [21].

The main concerns of human and wildlife exposure to PAEs are the potential adverse effects on reproduction, including problems with fertility, the development of newborns, and carcinogenicity [22]. PAEs can enter the human body through inhalation, ingestion and dermal absorption [23–24]. PAEs may potentially affect human testicular dysgenesis syndrome, reproductive development [25] and sex reversal [2]. PAEs acting as endocrine disruptors may contribute to many health problems such as hepatomegaly, osteoporosis, feminization of boys, weight loss, and skin and breast cancer [2]. Because PAEs can bioaccumulate over long term exposure, humans are at higher risk following continued consumption of contaminated water or food [5].

There were many studies on PAE concentration in landfill leachate conducted in EU and around the world [4, 7, 10, 13] in the past. Unfortunately no exhaustive studies on this topic have yet been conducted in Poland. Our research presents the first data on PAE concentrations in MSW landfill leachate. The aim of this study was to determine the concentrations of PAEs in the leachate from the chosen MSW landfills in Poland. Raw leachate was sampled four times over the period of one year in 2015/16.

## Materials and methods

### Characteristics of sampling sites and location

In 2015 five MSW landfills were chosen for phthalates analysis in raw leachate. All the landfills were located in Masovia Voivodship in central Poland, close to the capital city Warsaw. The landfills chosen for the analysis differ in their age, size and operation (closed or still in use). All the landfills were or are used for landfilling of non-hazardous and inert waste. The following landfills were chosen for sampling:

Landfill no. 1 is the largest sampled landfill which was opened in 1978. In 1996 a remediation process started, and landfill operations were terminated in 2011. The landfill contains around 5.5 million m^3^ of waste. The leachate has a collection system inside the landfill block, and a vertical barrier for controlling the flow. Leachate is collected in two roofed tanks.
Landfill no. 2 is the second largest landfill, which was opened in 1965. In 2009 one quarter was terminated and remediated, while another block is still operating and has a synthetic bottom isolation. The landfill contains around 2.5 million m^3^ of waste. The leachate collecting system is functioning inside the landfill blocks. Leachate is collected in roofed tank.
Landfill no. 3 was opened in 1970. In 2006 one quarter was terminated and remediated, while another part is still operating. The landfill contains around 960 thousand m^3^ of waste. The leachate collecting system is functioning inside the landfill block. Leachate is collected in an unroofed tank. The landfill has a synthetic bottom isolation.
Landfill no. 4 was opened in 1970 and is still operating. The landfill contains around 908 thousand m^3^ of waste. Leachate is collected in two large unroofed tanks. There is a vertical barrier for controlling the flow.
Landfill no. 5 is the smallest sampled landfill. It was opened in 1970 and is still operating. The landfill contains 832 thousand m^3^ of waste; it has natural clay isolation on the bottom. Leachate is collected in roofed tank.

All the landfills have systems for controlling and collecting leachate in storage tanks. The leachate is transported to sewage treatment plants.

**Fig 1:**
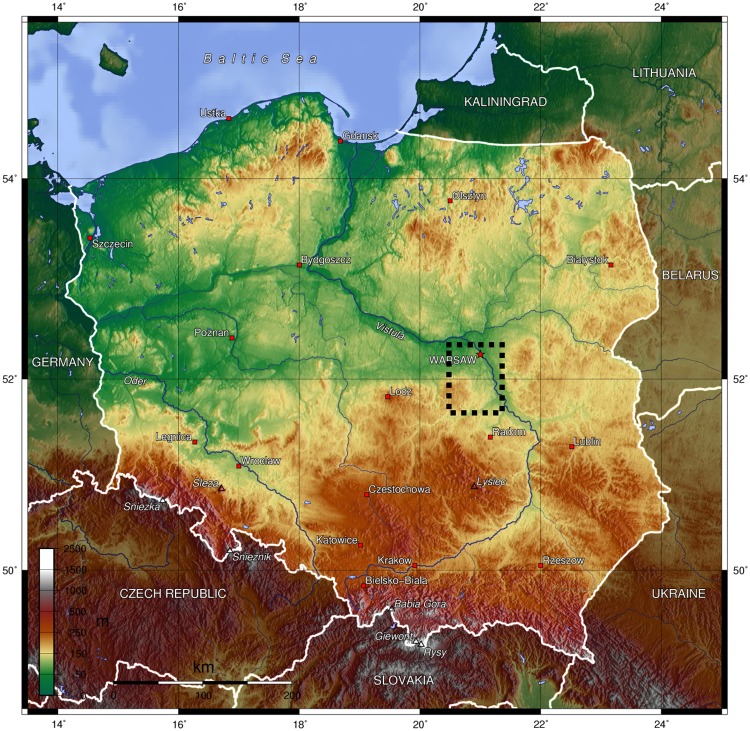
The location of the sampled landfills.

The locations of landfills are presented in Fig 1. The precise names and locations of the landfills sites are confidential. The landfills are labeled 1–5, where 1 is the largest and 5 the smallest in size.

### Materials

The following PAEs were chosen for analysis in this study, based upon historical popularity and high frequency of use: Dimethyl phthalate (DMP), Diethyl phthalate (DEP), Di-n-propylphtalate, Di-n-butyl phthalate (DBP), Di-isobutylphthalate (DIBP), Di-pentylphthalate(DPP), Butyl benzyl phthalate (BBP), Di-cyclohexylphthalate (DCP), Di (2-ethylhexyl)phthalate (DEHP) and Di-n-octylphthalate (DOP).

### Sampling of raw leachate

Leachate samples were collected four times over the period of one year in 2015/16 from five MSW landfills. Sampling took place in each of the four seasons to examine seasonal variation in PAE concentrations. The samples were collected directly from raw leachate tanks, according to guidelines in the Landfill manual [26]. The tanks were not purged, but three discrete grab or pumped samples were collected by subsurface sampling. In the case of grab samples, whenever possible individual samples were taken at differing locations and depths across and within the raw leachate tanks. To avoid cross contamination no plastic materials were used during sampling, transportation or analysis. Glass jars with an aluminium foil under the cap and a stainless steel bucket were used during the study. All jars were rinsed with leachate before sample collection. The color of the samples varied from yellowish to dark brown. Samples were collected in June, October and December of 2015, and in April of 2016. Overall there were 60 samples collected and analyzed in this study. Weather details and times of sampling are presented in Table 1.

**Table 1:** Details of the sampling times and weather condition.

Season of sampling	Date of sampling	Weather conditions	Average precipitation in the region* [mm]
Summer	30.06–01.07.2015	25°C, no precipitation, sunny and dry.	32,6
Autumn	09–10.10.2015	3–15°C, no precipitation, cloudy and dry.	47,6
Winter	14–16.12.2015	2°C, no precipitation, cloudy, precipitation two days before the sampling.	37,8
Spring	04–05.04.2016	10–15°C, no precipitation, sunny, precipitation three days before the sampling.	37,2

*based on the data from meteorological stations provided by the Institute of Meteorology and Water Management—National Research Institute (IMGW-PIB)

### Method of the analysis

PAE analysis was conducted using the internally developed laboratory method W-PTHGMS01 (based on US EPA 8061A, 3500, 3510) in an accredited laboratory (The Certificates of Accreditation no. 819/2015 and 319/2016). This method was developed for the analysis of drinking, waste or ground water matrices. To 500 mL of sample an extraction standard FTA-ISTD10 of acetone was added. The sample was then extracted twice with 30 ml dichloromethane. The extract was then transferred into hexane and concentrated to 0.25 ml. 1 μl of prepared solution was analysed. Gas chromatography coupled with mass spectrometry (model Agilent 7890/5975C) was used for the analysis. The sample was injected in splitless mode at 250°C. Separation was performed on the column at DB-5MS: 20 m (length), 0.18 mm (diameter), 0.18 μm (thickness of a phase in the column), ion m/z 149. 5-point calibration was used in the range of 0.5–10 μg/ml.

The following standards were used for the calibration:

ISTD     10 mg/ml, custom mix of deuterate phthalates, Chiron S-4727-10K-AC
LCS    2/20 mg/ml, phthalate standard 12 components, Absolute standards, 97625
Calibration 2/20 mg/ml, organic standard solution, Chromservis, 3389.20 K. A. 1.5 syringe std. neat; BBP-D4, Dr. Ehrenstorfer, 16168010.

It needs to be pointed out that landfill leachate is rich in dissolved organic matter, inorganic compounds, heavy metals and xenobiotic organic materials; therefore PAE analyses are complex, with a number of potential difficulties such as matrix interference. For all measured values measurement uncertainty was (+/-35%). The limit of quantification (LOQ) for each PAE (except DEHP) was 0.6 μg/L; the LOQ for DEHP was 1.3 μg/L. For some of the samples the LOQ was raised and in a few cases the value was not detected (Table 2).

**Table 2:** PAEs concentrations in Municipal Solid Waste (MSW) landfill raw leachate—summer, autumn, winter 2015 and spring 2016.

Compound name	Season	Content of phthalates (range (n = 3) in μg/L) in leachate in studied locations.				
		Landfill1	Landfill 2	Landfill 3	Landfill 4	Landfill 5
Dimethyl phthalate (DMP)	Summer 2015	<5.0	<5.0	<5.0	<2.5	<5.0
	Autumn 2015	<1.0	<1.0	<1.0	<1.0	<0.6
	Winter 2015	<2.5	<2.5	<0.6	<2.5	<0.6
	Spring 2016	<0.6	<0.6	<0.6	<0.6	1.08–1.98
Diethyl phthalate (DEP)	Summer 2015	<5.0	<5.0	<5.0	<2.5	<5.0
	Autumn 2015	<1.0	<1.0	<1.0	<1.0	<0.6
	Winter 2015	<2.5	<2.5	<0.6	<2.5	4.3–4.72
	Spring 2016	<0.6	0.61–1.08	<0.6	<0.6	1.64–1.84
Di-n-propyl phthalate	Summer 2015	<5.0	<5.0	<5.0	<2.5	<5.0
	Autumn 2015	<1.0	<1.0	<1.0	<1.0	<0.6
	Winter 2015	<2.5	<2.5	<0.6	<2.5	<0.6
	Spring 2016	<0.6	<0.6	<0.6	<0.6	<0.6
Di-n-butyl phthalate (DBP)	Summer 2015	<5.0	<5.0	<5.0	<2.5	<5.0
	Autumn 2015	ND	<1.0	<1.0	<1.0	<0.6
	Winter 2015	<2.5	<2.5	<0.6	<2.5	1.1–1.32
	Spring 2016	<0.6	<0.6–0.71	<0.6	<0.6	<0.6–1.08
Di-isobutyl phthalate (DIBP)	Summer 2015	<5.0	<5.0	<5.0	<2.5	<5.0
	Autumn 2015	<1.0	<1.0	<1.0	<1.0	<0.6
	Winter 2015	<2.5	<2.5	<0.6	<2.5	<0.6
	Spring 2016	<0.6	<0.6	<0.6	<0.6–1.13	1.28–2.17
Di-pentyl phthalate (DPP)	Summer 2015	<5.0	<5.0	<5.0	<2.5	<5.0
	Autumn 2015	<1.0	<1.0	<1.0	<1.0	<0.6
	Winter 2015	<2.5	<2.5	<0.6	<2.5	<0.6
	Spring 2016	<0.6	0.95–2.59	0.62–0.74	<0.6	<0.6
Butyl benzyl phthalate (BBP)	Summer 2015	<5.0	<5.0	<5.0	<2.5	<5.0
	Autumn 2015	<1.0	<1.0	<1.0	<1.0	<0.6
	Winter 2015	<2.5	<2.5	<0.6	<2.5	<0.6
	Spring 2016	<0.6	<0.6	<0.6	<0.6	<0.6
Di-cyclohexyl phthalate (DCP)	Summer 2015	<5.0	<5.0	<5.0	<2.5	<5.0
	Autumn 2015	<1.0	<1.0	<1.0	<1.0	<0.6
	Winter 2015	<2.5	<2.5	<0.6	<2.5	<0.6
	Spring 2016	<0.6	<0.6	<0.6	<0.6	<0.6
Di(2-ethylhexyl) phthalate (DEHP)	Summer 2015	32.2–38.6	64.9–73.9	ND	<5.0–19.3	ND-34.4
	Autumn 2015	ND—20.2	9.1–58.1	<5.8	<1.6	ND
	Winter 2015	16.5–19.6	34.4–52.6	2.2–2.5	<2.5	2.0–2.7
	Spring 2016	2.7–4.7	34.4–43.1	5.5–10.5	<1.3–2.3	3.6–50.5
Di-n-octyl phthalate (DNOP)	Summer 2015	<5.0	<5.0	<5.0	<2.5	<5.0
	Autumn 2015	<1.0	<1.0	<1.0	<1.0	<0.6
	Winter 2015	<2.5	<2.5	<0.6	<2.5	<0.6
	Spring 2016	<0.6	<0.6	<0.6	<0.6	<0.6

LOQ for all PAEs (except DEHP) was 0.6 μg/L, LOQ for DEHP was 1.3 μg/L. For some samples LOQ was raised and in few cases the value was not detected (ND) due to matrix interference.

Measurement uncertainty was (+/-35%).

## Results

### PAE concentrations in raw leachate

**Fig 2:**
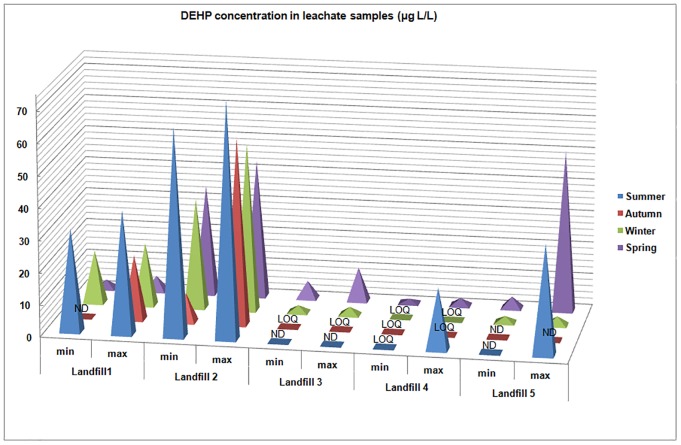
DEHP concentration in raw leachate samples (μg/L).

The results of PAE concentrations in raw leachate are presented in Table 2. All PAE concentrations (except DEHP) were below LOQ, or if were present, were in low concentrations. Among analyzed PAEs, DEHP was the most prevalent, and in concentrations often may times higher than other PAEs: ranging from <5.0 to 73.9 μg/L in summer 2015; <1.6 to 58.1 μg/L in autumn 2015; 2.05 to 52.6 μg/L in winter 2015 and <1.3 to 50.5 μg/L in spring 2016. The highest concentrations of DEHP were found in summer 2015 on landfill no. 2 (73.9 μg/L) and landfill no. 1 (38.6 μg/L). In spring 2016 the highest DEHP concentrations were detected on landfill no. 5 (50.5 μg/L). Moreover high concentrations of DEHP were detected on landfill no. 2 during all seasons (Fig 2).

DEHP concentrations fluctuated in the leachate among the landfills, whereas the other PAEs showed no significant differences among the different sites.

In winter 2015 DEP and DBP were detected on landfill no. 5. DEP concentrations were between 4.3 and 4.72 μg/L, and DBP ranged from 1.1 to 1.32 μg/L. In spring 2016 four PAEs were detected in leachate from three landfills. DMP was detected on landfill no. 5 with concentrations between 1.08 and 1.98 μg/L. DEP was found on landfill no. 2 with concentrations from 0.61 to 1.08 μg/L, and on landfill no. 5 from 1.64 to 1.84 μg/L. DBP was also found in landfills no. 2 and 5, with concentrations ranging from <0.6 to 0.7 μg/L, and <0.6 to 1.08 μg/L respectively. DIBP was detected on landfill no. 4 and 5, with concentrations between <0.6 to 1.13 μg/L and 1.28 to 2.17 μg/L respectively. All the other analyzed PAEs (except DEHP) in summer and autumn 2015 were not detected or were below LOQ.

### Seasonal changes

The highest concentrations of DEHP were observed on both largest landfills in spring 2015, lower in summer and autumn; and the lowest in winter 2016. As for the other landfills this trend was not observed. It needs to be noted that the accumulated precipitation in Warsaw [27] in 2015 was unusually low (396.3 mm) in comparison to that in 2014 (511.2 mm) or in 2013 (618.1 mm). This fact may affect PAE concentrations.

## Discussion

MSW landfills’ leachates are directed and treated in municipal Sewage Treatment Plants (STP) in Poland. In all the tested landfills the leachates were collected in raw leachate tanks (three roofed, and two unroofed) and then transported to STPs by trucks. Leachates are handled and processed the same way on all the tested landfills, none being recirculated to landfill blocks.

The highest concentrations of all PAEs were found for DEHP. Similar observations were reported by others [4, 11, 10, 28]. The results are as expected, as historically until present, DEHP was the most used plasticiser, and represented half of the total PAE consumption in Western Europe [10].

Taking into consideration the seasonal changes of DEHP concentrations, the highest concentrations of DEHP found in each landfill (except landfill no. 5) were in summer 2015. It potentially is explained by the fact that summer is the driest season of the year and the leachate was the least diluted. High concentrations of DEHP were observed in landfill no. 2 irrespective of season. This result is perhaps explained by the fact that this landfill is the largest of the five studied sites, which remains in operation, thus containing the most waste, including plasticizers. In a Japanese study two municipal waste landfills with leachate collection systems (and leachate tanks) were analysed, resulting in no observable seasonal changes in DEHP concentrations [11].

There is a general correlation between DEHP concentrations and landfill size [Fig 2], with the exception of landfill no. 5. The correlation can be explained by more waste and plasticizers being released into leachate by a larger site, with comparable precipitation for each site. The anomaly of landfill no. 5 (the smallest of all the studied landfills) might be attributed to the fact that it had the least advanced waste management practices and contained unknown or unconfirmed waste sources.

The resultant DEHP concentrations are comparable to data from studies from Finland [1–89 μg/L], Japan [9.6–49 μg/L], China [n.d.-46 μg/L], Sweden [<1–9 μg/L], Denmark [<1–3 μg/L] and Thailand [65.6 μg/L] (Table 3). Our measured DEHP concentrations were lower than those measured in Sweden (Göteborg) [97–346 μg/L], Germany [up to 240 μg/L] and Italy [88–460 μg/L]. It could be explained by the different compositions of waste, mainly the amounts and types of plasticizers historically used in Western and Eastern Europe. Another reason might be the time when the studies were conducted; all of those studies were performed more than 15 years ago, when the use of DEHP was higher than present.

**Table 3:** Occurrence of phthalates in MSW landfill leachate—based on the data presented in literature (μg/L).

Sampling site	Sampling date	DMP	DEP	DBP	DIBP	BBP	DEHP	DNOP	Reference
11 landfills in Finland	1998–2001	ND-<1	n.d-9	<1–17	-	ND- 1	1–89	ND-<1	[29]
2 landfills in Japan	4 times in 2000/2001	-	1–8.4	3–15	-	0.7–7.8	9.6–49	-	[13]
2 landfills in Shanghai, China	04-07/2007	ND	ND	71–80	ND	ND	40–46	-	[31]
1 landfills in Wuhan, China	12/2007	ND-43.2	ND	ND-15.1	-	ND	ND-7.2	ND	[7]
3 landfills in Göteborg, Sweden	-	-	-	-	-	-	97–346	-	[30]
Bavaria, Germany	-	-	-	-	-	-	26.4–240	-	[30]
7 landfills/landfill cells in Sweden	08/1998	<1	<1–11	<1–23	-	<1–5	<1–9	-	[10]
6 landfills in Denmark	02/1999	<1	<1	<1	-	<1–2	<1–3	-	[10]
2 landfills in northern Germany	02/1998	-	11–13	4–19	-	<1–7	<1-≤ 20	-	[10]
2 landfills in Italy	09/1998	<1	7–27	6–10	-	<1	88–460	-	[10]
Thailand	-	20.8	12.5	35.4	-	21.5	65.6	-	[4]

where: “ND”- not detected, “-“- no data/not analized,

Our data show that all other PAE concentrations were mostly below LOQ, or if present (in 11 cases out of 180) were in low concentrations (<0.6–4.7 μg/L). There are a number of similarities across studies (Table 3), though there are exceptions where much higher concentrations have been found (such as China and Thailand, where DBP concentrations were as high as 80 μg/L and 35.4 μg/L respectively). A lot of factors could influence those differences, mainly the amounts and types of plasticizers directed to those landfills.

It was found in the study that although landfill no. 1 underwent remediation in 1996 and its operations were terminated in 2011, leachate still contained a significant concentration of DEHP. This could be attributed to an expectedly slow mineralization of DEHP under anaerobic conditions that are prevalent in a landfill [29]. DEHP with a log Kow of about 7.5 is lipophilic and is expected to strongly adsorb to organic matter, and soil or rock media, such as accumulates in municipal landfills [7, 30]. DEHP is expected to be released from waste for a long time [13]. Those facts were confirmed by our results: DEHP emissions are still accruing many years after closing the landfill. On the same landfill no other PAEs were measured in the leachate, including DBP, which was the second most used plasticizer after DEHP in Poland (its production stopped around 2004). It could be suggested that other PAEs already leached out from the waste or were biodegraded to below LOQ levels.

As no data were available on former levels of PAEs in the studied areas, no conclusion can be made concerning long term changes in PAE levels in landfill leachate.

## Conclusions

PAEs were detected in leachate samples from each of five studied landfills. The following PAEs were detected in at least one sample: DEHP, DEP, DMP, DBP, DIBP.

Out of all ten PAEs DEHP was predominant, with concentrations ranging from <LOQ (smaller landfills) up to 73.9 μg/L (large landfill). DEHP was present in 65% of samples (100% in spring, 80% in winter, and 40% in summer and autumn samples).

In 25% of samples were DEHP concentrations below the acceptable EU limit for surface water (1.3 μg/L), while 75% of samples were 1.7 to 56 times higher than that value.

In 55% of samples were DEHP concentrations below the EU and WHO drinking water limit (8 μg/L), while 45% of samples were 1.3 to 9.2 times higher than that value.

At the two largest landfills, including the landfill which has been remediated and closed for last five years, the presence of DEHP was observed in each of the four seasons,.

PAEs (and in particular DEHP) are ubiquitous environmental contaminants present in landfill leachate.
